# Effect of various surface coating methods on surface roughness, micromorphological analysis and fluoride release from contemporary glass ionomer restorations

**DOI:** 10.1186/s12903-024-04234-5

**Published:** 2024-04-29

**Authors:** Mohanad H. Alebady, Hamdi H. Hamama, Salah H. Mahmoud

**Affiliations:** Conservative Dentistry Department, Faculty of Dentistry, Mansoura University, Mansoura, 35516 Egypt

**Keywords:** Glass Ionomer cement, Resin modified glass ionomer cement, Surface roughness, Atomic force microscopy, Micromorphological analysis

## Abstract

**Objective:**

To evaluate the effect of various surface coating methods on surface roughness, micromorphological analysis and fluoride release from contemporary resin-modified and conventional glass ionomer restorations.

**Materials & methods:**

A total of 72 permanent human molars were used in this study. The teeth were randomly assigned into 2 groups according to type of restorative materials used; resin modified glass ionomer cement and conventional glass ionomer (SDI Limited. Bayswater Victoria, Australia). Each group was subdivided into 3 subgroups according to the application of coat material; Sub-group1: without application of coat; Sub-group2: manufacturer recommended coat was applied and sub-group3: customized (vaseline) coat was applied. Each group was then subdivided into two divisions according to the time of testing; immediate (after 24 h) and delayed (after 6 months of storage). Three specimens from each sub-group were selected for surface roughness test (AFM) and another 3 specimens for the micromorphological analysis using scanning electron microscope (SEM). For the fluoride release test, a total of 60 cylindrical discs were used (*n* = 60). The discs were randomly split into 2 groups according to type of restorative materials used (*n* = 30); resin modified glass ionomer cement and conventional glass ionomer. Each group was subdivided into 3 subgroups (*n* = 10) according to the application of the coat material; Sub-group1: without application of coat; Sub-group2: with the manufacturer recommended coat and sub-group3: with application of customized (vaseline) coat. Data for each test was then collected, tabulated, were collected, tabulated, and tested for the normality with Shapiro-Wilk test. Based on the outcome of normality test, the significant effects of variables were assessed using appropriate statistical analysis testing methods.

**Results:**

Regarding the data obtained from surface roughness test, Shapiro-Wilk test showed normal distribution pattern of all values (*p* > 0.05). Accordingly, Two-way ANOVA outcome showed that the ‘type of restoration’ or ‘test time’ had statistically significant effect on the AFM test (*p* < 0.05). Regarding Fluoride specific ion electrode test 2-way ANOVA followed by Least Significant Difference (LSD) Post-hoc test revealed significant difference among the groups (*p* < 0.05). It showed that SDI GIC group after 14 days of measurement had the highest mean of fluoride release (36.38 ± 3.16 PPM) and SDI RMGIC after 30 days of measurement had the second highest mean of fluoride release (43.28 ± 1.89 PPM). Finally, regarding the micromorphological analysis using SEM, a slight difference was observed between the studied groups.

**Conclusions:**

Based on the results of this study, various coatings enhance surface roughness in the initial 24 h of restoration insertion. Different coat types seems that have no influence on fluoride release and the micromorphological features of the restoration/dentin interface.

## Introduction

Glass ionomer cements were introduced as a restorative material that adhere to tooth substrate with minimal adverse effect on the pulp. Glass ionomer cement is a substance which has the ability to chemical bond with calcium ions in hydroxyapatite. Despite of it´s low-cohesive strength, compared to resin-based composite, it bonds to both sound and caries-affected tooth dentin. GICs exhibits an anticariogenic effects and this might attributed to its fluoride release [1]. Glass ionomer restorations showed a potent antimicrobial properties particularly on oral bio-film level [1]. The esthetics of GICs are expected to evolve further. For example, with the inclusion of novel polymer and surface chemistries that allow GICs to resist color change even when exposed to staining media such as beverages or certain foods, the color change over time may be decreased. RMGICs currently have the same initial translucency and optical characteristics as leading composite resin materials. To follow composite resins, optical qualities such as translucency and opacity values, as well as nearer color matching, may be improved. Although advances are being made, traditional-cured GICs still lack the translucency qualities of contemporary composites [2]. 

Conventional GICs are characterized by water-sensitivity during the setting process. This short-term sensitivity can have a negative impact on the material’s mechanical qualities as well as its appearance [3]. Water sorption and solubility affect surface roughness of restoration by affecting the physical, optical, and thermodynamic characteristics of all restorative materials [4]. This is one of the elements that should be highlighted, especially for GIC, which has a long setting reaction time. During the setting process, water loss can cause micro fissures in the restoration’s structure, volumetric alterations, and adhesion weakness. Surface erosion occurs when calcium and aluminium ions are lost from the restoration’s surface due to early contact with moisture, and it reduces the restoration’s translucency [4]. 

In light of frequent exposure to acids from the biofilm, current GICs achieve an equilibrium of low ion release, which may not be enough to prevent demineralization of tooth structure [5]. To enable more dependable anti-cariogenic effects, research on existing materials has been done [5]. Modified glass ionomer has been introduced. While GICs are cross-linked polyacid matrix in which fillers are the glass particles in the cement, in RMGIC the matrix also contains a polymer network of resinous materials throughout the set cement [1]. The only difference is that the latter contains a polymerizable resin. RMGICs have a higher toughness and better esthetics than conventional glass ionomer cement [1]. The homogeneous dispersion of ultrafine highly reactive glass particles and the increase in the molecular weight of the polyacrylic acid reinforce this GIC [6]. Improved mechanical qualities distinguish RMGIC. This type of GIC, according to the manufacturer, can be employed in loadbearing posterior approximal restorations as well as occlusal and cervical restorations. In comparison to earlier conventional GICs [6]. This improved GIC has higher flexural strength, surface hardness, and compressive strength, according to later research. These characteristics may allow this material to be employed in a variety of clinical dentistry applications [6]. 

The recommendation has been to coat the GIC’s surface to overcome the problem of water sensitivity. As long as this coat is on the restoration, the mechanical qualities are preserved. Recently, a new resin coating for traditional GICs has been created, which is distinguished by the incorporation of nanofiller particles to improve the GIC’s marginal sealing and wear resistance. Several laboratory investigations have shown that this covering improves fracture strength while also minimizing early wear [5]. The application of self-adhesive coating and ageing influenced the load-bearing capacity of the restorative materials. The coated glass ionomer cement (CGIC) was much harder than the RMGIC and resin composite, but had significantly lower flexural strength. The wear resistance was not greatly improved by a resinous coat. Under dry conditions, resin coating enhanced the flexural strengths of GICs. For all glass ionomer restorations, a protective covering, such as G-coat plus or light polymerized low viscosity unfilled resin adhesives, is required to strengthen the wear resistance of the restorative materials [7]. Accordingly, this study was designed to evaluate the effect of various surface coating methods on surface roughness, micromorphological analysis and fluoride release from contemporary resin-modified and conventional glass ionomer restorations before and after storage. The null hypotheses of this study were that there is no significant effect of different surface coating methods on (I) surface roughness (II) fluoride release of resin-modified compare to conventional glass ionomer restorations.

## Materials and methods

This laboratory study was done using two commercially available resin-modified glass ionomer (RIVA LC, SDI) and conventional glass ionomer cement (RIVA conventional glass ionomer cement, SDI) with its manufacturer recommended coat (RIVA LC coating material, SDI) and customized (vaseline) coat. The commercial name, manufacturer, composition, and application guidelines for the material are provided in (Table 1).

**Table 1:** Materials used in this study

Materials	Manufacturer	Composition	Applications	Batch number
Resin modified glass ionomer cement (RMGIC) SDI RIVA light cure	(SDI Limited. Bayswater Victoria, Australia)	Compartment 1: Acrylic acid homopolymer (15–25%), 2-hydroxyethyl methacrylate (15–25%), dimethacrylate cross-linker (10–25%), acid monomer (10–20%), tartaric acid (5–10%) Compartment 2: Glass powder (93–100%)	1. Push the plunger. 2. Place the capsule into the amalgamator 3. Mix for 10 s 4. Place capsule into the riva capsule applicator 5. Click the trigger o until RMGIC is seen through the clear nozzle 6. Light cure for 10 s after placement of RMGIC into cavity	J2108055EA
Conventional glass ionomer cement (GIC) SDI RIVA self cure		Fluoroaluminosilicate glass, acrylic monomer, and polyacrylic acid + tartaric acid	1. Mixing on mixing pad with an agate spatula. 2. Powder/liquid are used as recommended by the manufacturer. 3. The resultant mixture should have a glossy surface.	11,954,761
Light cured coating material RIVA coat		Acrylic monomer	1. Placement of GIC/RMGIC according to manufacturer’s instructions. 2. Apply Riva Coat to all exposed surfaces of restoration 3. Light cure for 20 s. 4. Replace cap immediately after use.	210,274
Vaseline® Brand	Unilever, United Arab Emirates	Petrolatum, BHT and Tocopheryl Acetate	1. Place the glass ionomer cement according to manufacturer’s instructions. 2. Apply vaseline to all exposed surfaces of restoration.	67,920,919
Artificial saliva	Prepared in department of Pharmaceutics, Faculty of Pharmacy, Mansoura University [8]	1. Carboxyl methyl cellulose sodium salt low viscosity (Na CMC). 2. Magnesium chloride hexahydrate(Mgcl_2_.6H_2_O). 3. Methyl P-hydroxy benzoic acid (methyl paraben ) 4. Potassium chloride (KCL). 5. Calciumchloride dihydrate (CaCl_2_.2H_2_O). 6.Potassium dihydrogen orthophosphate (KH_2_PO_4_). 7. Di-potassium hydrogen orthophosphate (K_2_HPO_4_). 8. Distilled water.	1. Replace every 48 h. 2. Should be place in the refrigerator to save artificial saliva from distortion. 3. Before each replacement, artificial saliva should be place at room temperature.	

### Teeth selection

Seventy-two permanent human molars were used in this study. The collected molars were extracted for therapeutic reasons unrelated to the study, with prior informed consent from healthy individuals who were seeking dental care at The Oral and Maxillofacial Surgery Department Clinic, Faculty of Dentistry, Mansoura University, Faculty of Dentistry at Mansoura University. The patients were voluntary donating the extracted teeth to The Faculty for utilizing in research purpose. The study protocol was approved from Faculty of Dentistry at Mansoura University, ethical approval number (A15080622). Teeth were thoroughly cleaned of calculus and soft tissue deposits before being rinsed with distilled water and a low-speed rubber cup with prophy paste using a hand scaler. Following that, teeth with pre-existing flaws, cracks, or restorations were excluded using a stereomicroscope (SZ TP, Olympus, Tokyo, Japan). Teeth were kept in 0.5% chloramine T solution for 72 h before being placed in deionized water, which was changed every 2 weeks.

### Specimen preparation

Molars were placed in polyvinyl chloride cylinders with 1.8-centimeter diameter and 2-centimeter height to fit the enormous roots of the molars. Auto-polymerizing acrylic resin was packed into these cylinders. Later, with the acrylic resin still in its doughy state, each tooth was vertically invested in the center of the cylinder with a metal ring. Two opposing screws were used to centralize the molar parallel to the long axis of an acrylic resin mold. The height of acrylic blocks were below the CEJ of the teeth.

A diamond saw (Isomet 4000, Buehler Ltd., Lake Bluff, IL, USA) was used to cut the specimens. Each tooth had its occlusal enamel and superficial dentin shaved away, revealing the dentin closer to its center. The dentin surface was ground using wet 600 grit silicon carbide sheets (SIA Brand, Switzerland) for 30 s to create uniform smear layers.

### Study grouping

Seventy-two molars were randomly assigned into 2 groups according to type of restorative materials used; resin modified glass ionomer cement or conventional glass ionomer cement. Each group was subdivided into 3 subgroups according to the application of coat material; Sub-group1: without application of coat; Sub-group2: manufacturer recommended coat was applied and sub-group3: Vaseline® Brand (Unilever, UAE) coat was applied.

Half of the specimens of each group were tested immediately, while the remaining halves were tested after 6 months of storage in artificial saliva at (37 ± 1ºC) in an incubator (BTC, Model: BT1020, Egypt). The storage medium was changed with a new fresh one every week.

Sample size calculation was based on cross-sectional hardness between treated arm versus control group retrieved from previous research [9]. Using G power program version 3.1.9.7 to calculate sample size based on effect size of 1.17 (127 ± 1.03 versus 126 ± 0.62) ,using 2-tailed test, α error = 0.05 and power = 80%, the total calculated sample size will be 13 in each group.

### Measurement of surface roughness using atomic force microscopy (AFM)

**Fig. 1:**
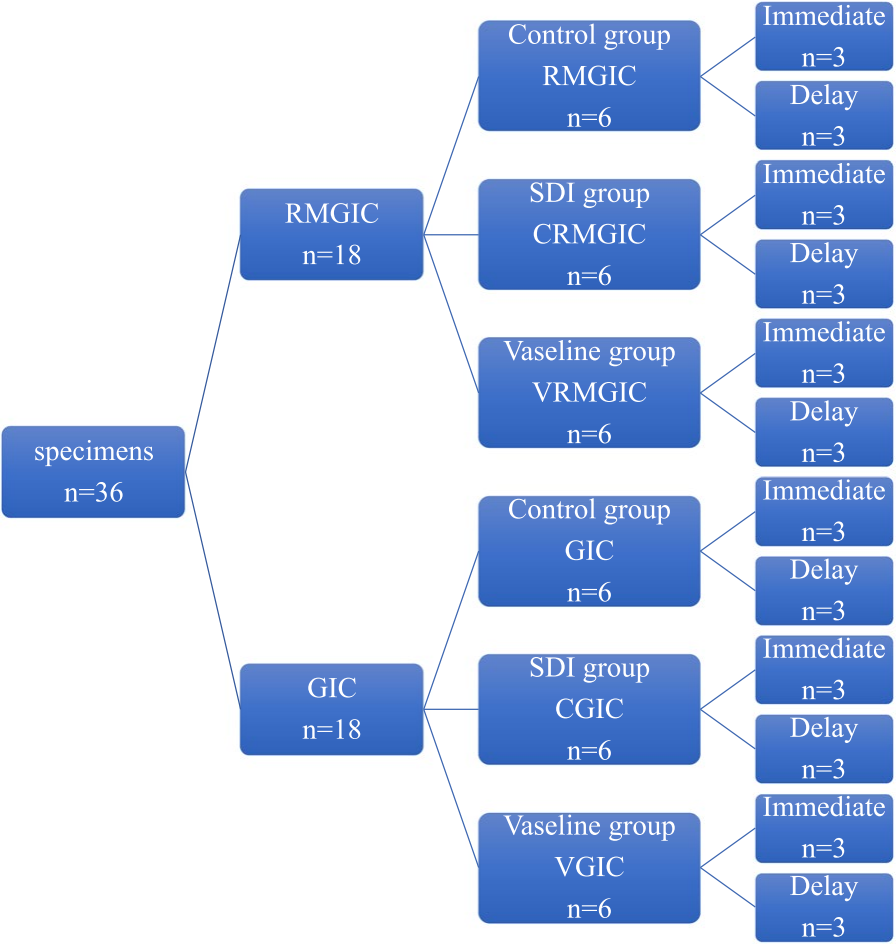
Diagram showing study design for surface roughness test

Three specimens were used in this test from each subgroup with a total number of thirty-six teeth (Fig. 1). The evaluation of surface roughness was carried out using AFM (Model. FleXAFM3). The scan area 10 × 10 Mm2 and number of data points: 256 × 256 at scan rate 1HZ. The AFM was operated in contact mode using nanoconductive silicon probe using Nanosurf C3000 (version 3.5.0.31) software. All measurements were performed at the middle one third of restoration surface for each tooth and the average was recorded. The results were obtained to compare restoration surface roughness before and after storage.

Micrographs were taken for each specimens using a digital microscopy with a built in camera linked to a computer using 120 X magnification. The micrographs were documented with a resolution of 1280 × 1024 pixels per image. The images were cropped to 350 × 400 pixel using Microsoft picture manager to standardize roughness areas, and analyzed using WSxM software. All limits, sizes, frames and measured parameters were expressed in pixels within the WSxM software. So, the system calibration was made to convert the pixels into absolute real world units, this made by comparing an object of known size with a scale produced by software. Consequently, 3-D image of specimens profile surface was constructed, then collected for each specimen, in the central area and in the sides at area of 10 Mm x 10 Mm. WSxM software was used to analyze surface roughness average expressed in Mm, which can be assumed as a reliable indices of surface roughness.

### Fluoride specific ion electrode test

Thirty test specimens of each GIC and RMGIC were formed into discs of 10-mm diameter and 2 mm thickness by using 3D dimension resin mold was specially designed with a central depression of the same dimension as the disc. All materials were handled according to the manufacturers’ instructions.

**Fig. 2:**
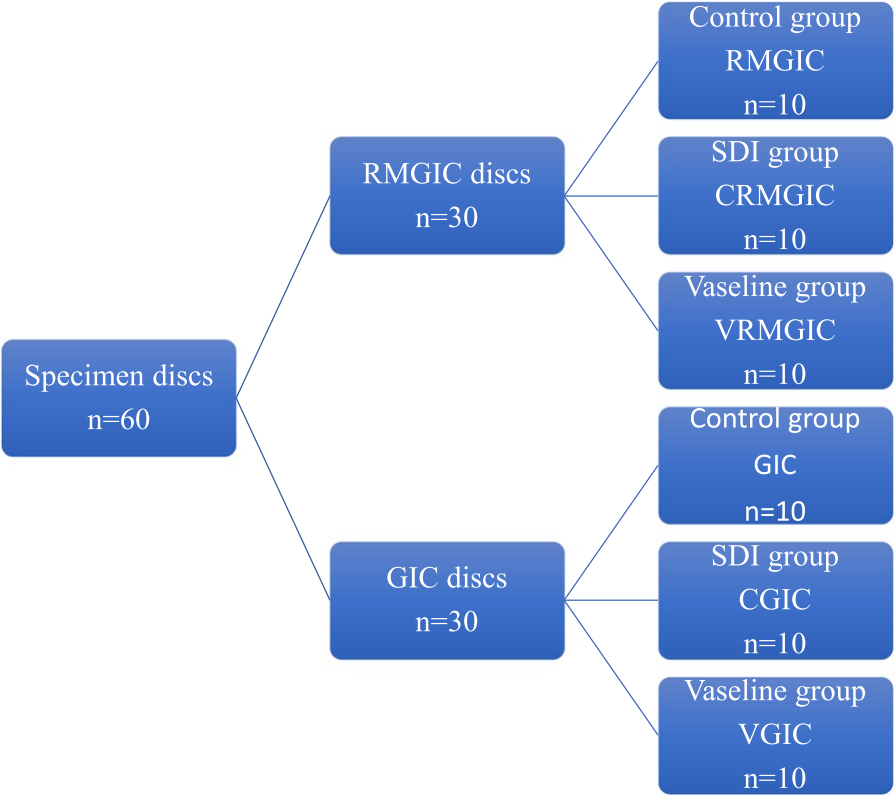
Diagram showing study design for fluoride release test

Each 30 discs subdivided in to 3 groups (*n* = 10), according to the type of coat placed for each one (no coat, SDI Riva coat or customized coat) (Fig. 2). For each coat group, coat was placed after complete setting of the material by using a dental brush (one layer for each disc). Each test specimen was immersed in artificial saliva in a sealed container stored in an incubator at 37 °C. The bottom of each container had a raised center that ensured that the sample disc was tilted to expose all of its surfaces to the artificial saliva. The fluoride levels in the solutions were measured in different time of storage (after 24 h, 7days, 14 days, 30 days, 45 days, 60 days ,75 days 90 days).

The test was conducted for each sub-group by taking 4 mm from the artificial saliva media where each sub-group has been stored. Five reading for each sub groups were recorded from At each storage time period. Fluoride ion was measured in artificial saliva using The SPADNS colorimetric method (SM 4500 F standard method for examination of water and waste water 24th edition 2023). The method is based on the reaction between fluoride and a zirconium-dye lake. Fluoride reacts with the dye lake, dissociating a portion of it into a colorless complex anion (ZrF6 2- ); and the dye. As the amount of fluoride increases, the color produced becomes progressively lighter.

The SPADNS reagent is prepared by dissolving 958 mg (0.958 g) SPADNS (Sulfanilic acid azochromotrop, Sigma Aldrich, USA) in deionized water and dilute to 500 ml deionized water. Zirconyl-acid reagent is prepared by dissolving 133 mg (0.133 g) zirconyl chloride octahydrate, ZrOCl2–8H2O (Sigma Aldrich, USA) in about 25 mL deionized water then by adding 350 mL conc HCl and dilute to 500 ml with deionized water. Acid zirconyl-SPADNS reagent is prepared by mixing equal volumes of SPADNS solution and zirconyl-acid reagent.

A group of 6 standard solutions with fluoride concentration 0.0, 0.2, 0.0.4, 0.6 ,0.8,1.0 ppm were prepared from a certified reference material of fluoride ( Merck KGaA, Darmstadt, Germany) and the absorbance was measured at 570 nm for the standards using UV-visible double beam spectrophotometer ( LAMDA 365, PerkinElmer, USA ).Samples then measured against the prepared standards after been diluted by ratio 1 : 25.

### Micromorphological analysis of restoration / tooth interface using scanning electron microscope (SEM)

For this test, the same study design of surface roughness test was used (Fig. 1). The immediate subgroups (3 teeth from each sub groups) were sectioned and tested after 24 h of restorative procedure. The delayed subgroups (3 teeth from each sub groups) were sectioned and tested after 6month of aging. This test used to obtain and quantitative data of tooth structure, which explaining the complex between restoration-tooth interface for evaluation of hybrid layer ultra-morphology.

The specimens were sectioned mesio-distally into two equal halves along the long axis of the teeth in a direction perpendicular to the restoration-dentin interface using a water-cooled diamond disc under low speed (IsoMetTM 4000, Buehler Ltd; Lake Bluff, IL, USA). Each half was grounded with coarse (600 grit), medium (800 grit), fine (1000 grit), and extra fine (1200grit, 2000grit) silicon carbide papers (SIA Brand Switzerland) under water. Final polish was obtained with fine diamond pastes with particle size (4 μm, 2 μm, 1 μm) respectively (ENA polishing system, Micerium S.p.A.) with a polishing piece of velvet fabric. Then, the first half of each tooth was cleaned in ultrasonic bath for 30 s (XH-E412 ultrasonic cleaner, Xinghua, China). The second half of each tooth was subjected to an acid\base challenge for characterization of the hybrid-like layer. This process include application of 10% orthophosphoric acid solution for 5 s on the tooth/restoration interface to demineralize dentin collagen fibers. Then 5% sodium hypochlorite solution was applied for 5 min to remove organic components. The specimens were prepared for scanning electron microscope (SEM) by being gold sputtered twice (SPI Module Sputter Carbon/Gold Coater, EDEN Instruments, Tokyo, Japan), and then observed with secondary electron detection mode using SEM (JSM-6510LV, JEOL, Tokyo, Japan) at an accelerating voltage of 30 KV and a working distance of 9–12 mm at magnification of ( 2000 x).

### Statistical analysis

All the data were collected, tabulated, and tested for the normality with shapiro-Wilk test. Based on the outcome of normality test, the significant effects of variables were assessed using appropriate statistical analysis testing methods. The threshold of significance was fixed at 5% level. The results were considered significant when *p*-value ≤ 0.05. Data analysis was performed by SPSS software, version 25 (SPSS Inc., PASW statistics for windows version 25. Chicago: SPSS Inc.).

## Results

### Measurement of surface roughness using AFM

**Fig. 3:**
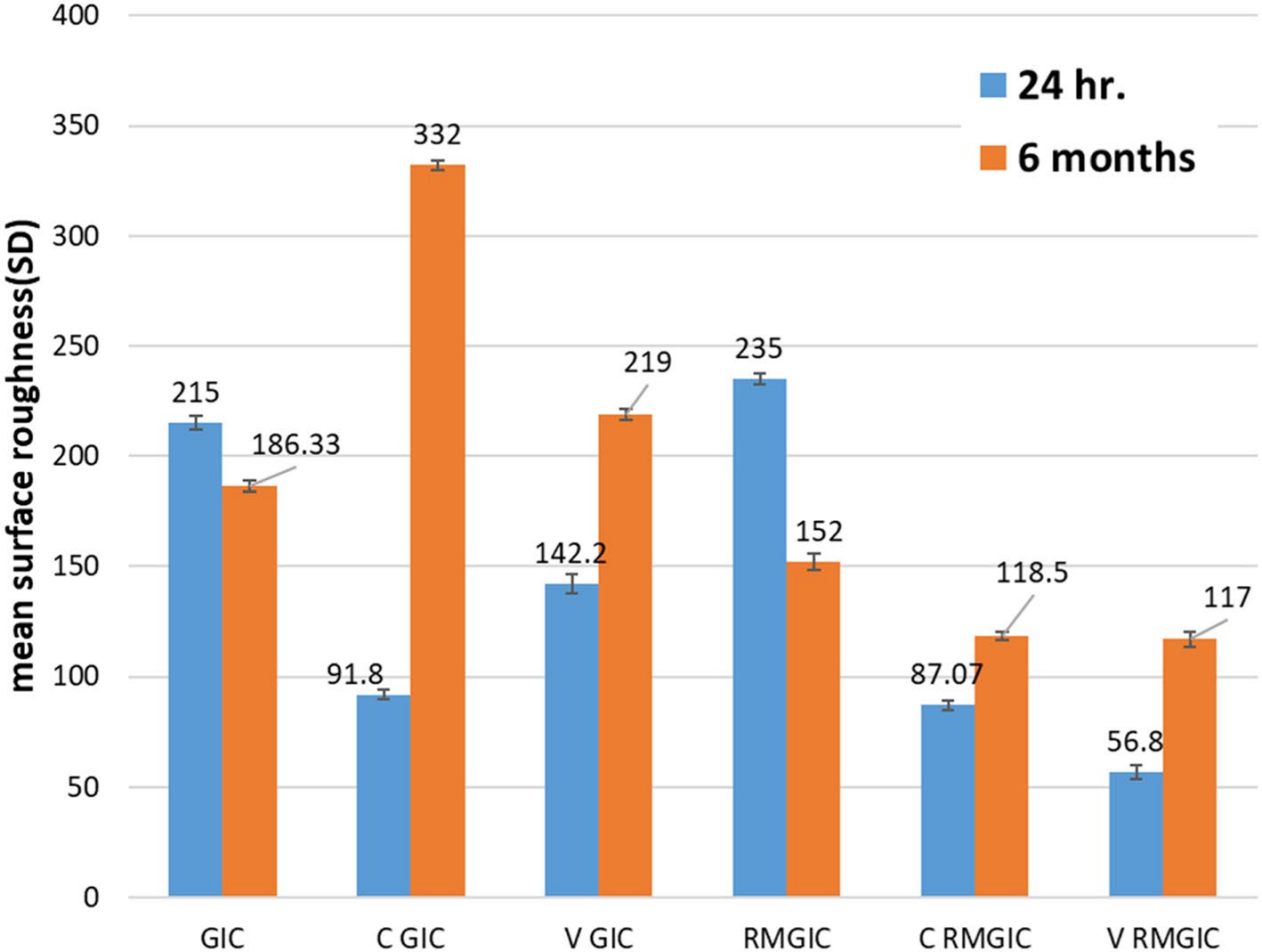
Mean surface roughness between studied groups at 24hours and 6 months

Regarding the data obtained from the AFM of all groups in this study, the results of Shapiro-Wilk test revealed normal distribution pattern of all values (*p* > 0.05). Consequently, Two-Way ANOVA to compare between the groups in immediate and delay storge time periods (Table 2). The outcome of 2-Way Anova followed by multiple comparison tests revealed that the mean surface roughness of GIC is slightly higher in immediate rather than delay. The mean surface roughness of CGIC is highest in delay as compered with all groups as well as with immediate result of CGIC group. The mean surface roughness of VGIC is higher in delay group rather than immediate group. The mean surface roughness of RMGIC is higher in the immediate group rather than delay group. The mean surface roughness of CRMGIC is slightly higher in the delay group rather than immediate group. The mean surface roughness of VRMGIC is higher in the delay group rather than immediate group (the mean surface roughness of VRMGIC in the immediate group is the lowest mean in all groups) (Fig. 3).

**Table 2:** Comparison of surface roughness at 24 h, 6 months between studied groups

Surface roughness average	A.GIC	B.C GIC	C.V GIC	D.RMGIC	E.C RMGIC	F.V RMGIC	Test of significance
**24 h.**	215.0 ± 3.1^BCDEF^	91.8 ± 2.3^ACDEF^	142.0 ± 4.5^ABDEF^	235.0 ± 2.5^ABCEF^	87.0 ± 2.1^ABCDF^	56.8 ± 3.1^ABCDE^	*F* = 15890.87 *P* < 0.001
**6 months**	186.3 ± 2.5 ^BCDEF^	332.0 ± 2.3 ^ACDEF^	219.0 ± 2.5 ^ABDEF^	152.0 ± 3.6 ^ABCEF^	118.5 ± 2.1 ^ABCDF^	117.0 ± 3.5 ^ABCDE^	*F* = 27240.66 *P* < 0.001
**Paired t test**	*P* < 0.001	*P* < 0.001	*P* < 0.001	*P* < 0.001	*P* < 0.001	*P* < 0.001	

Superscripted A denote significant difference with GIC, Superscripted B denote significant difference with C GIC, Superscripted C denote significant difference with VGIC, Superscripted D denote significant difference with RMGIC, Superscripted E denote significant difference with C RMIGIC, Superscripted F denote significant difference with V RMGIC group

**Fig. 4:**
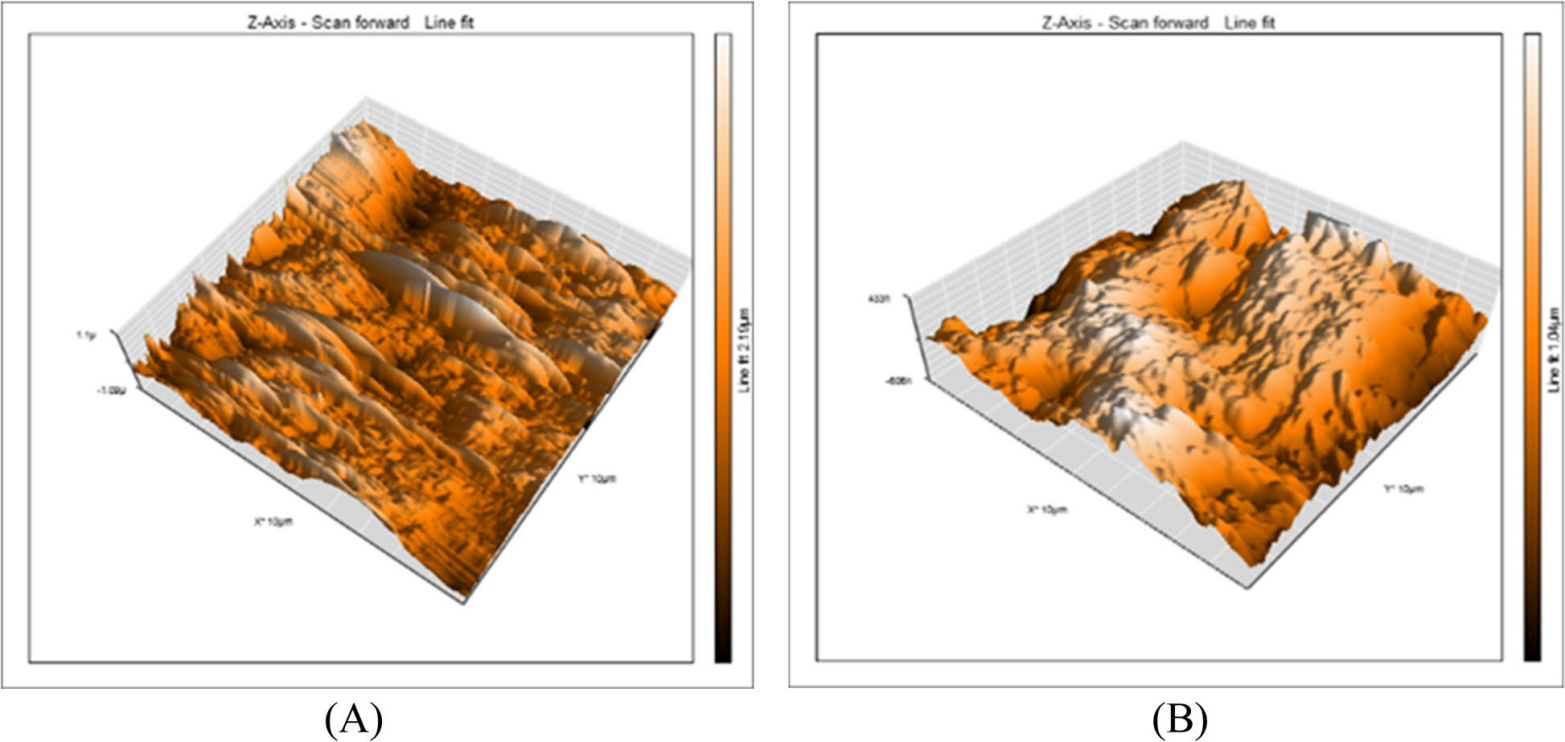
Three-dimension AFM contact mode micrographs of RMGIC after 24 hours (**A**) and 6 months (**B**) of restoration placement

**Fig. 5:**
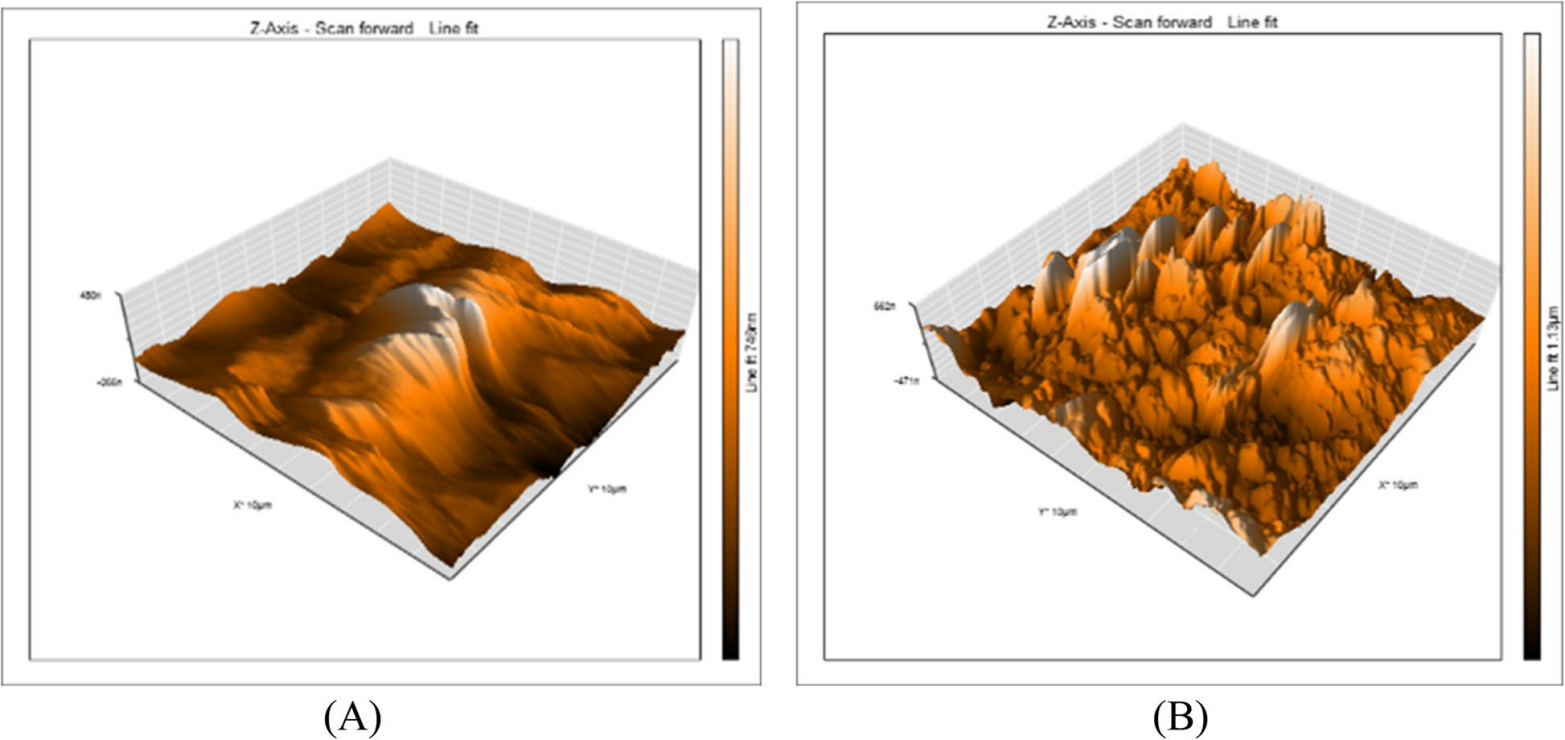
Three-dimension AFM contact mode micrographs of CRMGIC after 24 hours (**A**) and 6 months (**B**) of restoration placement

**Fig. 6:**
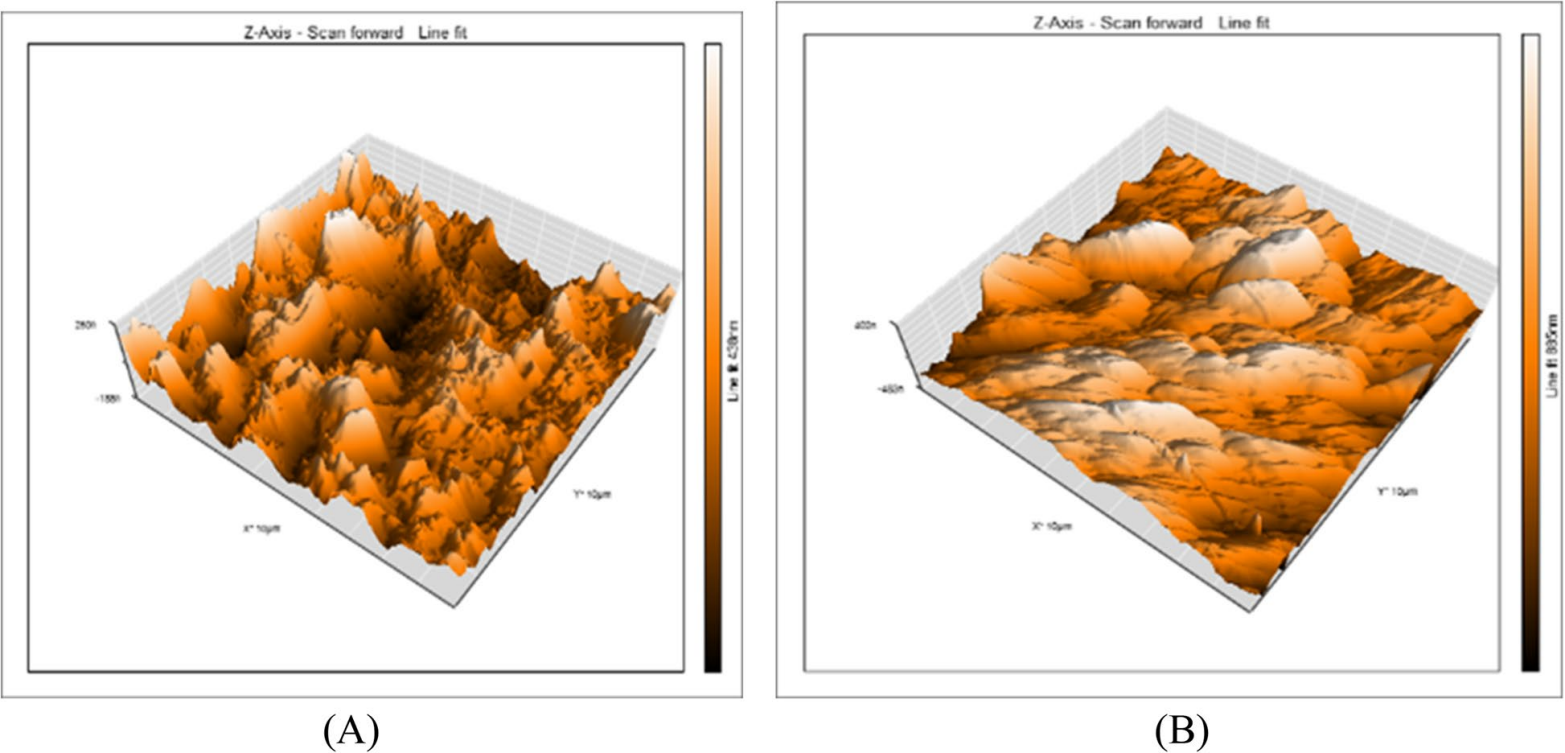
Three-dimension AFM contact mode micrographs of VRMGIC after 24 hours (**A**) and 6 months (**B**) of restoration placement

**Fig. 7:**
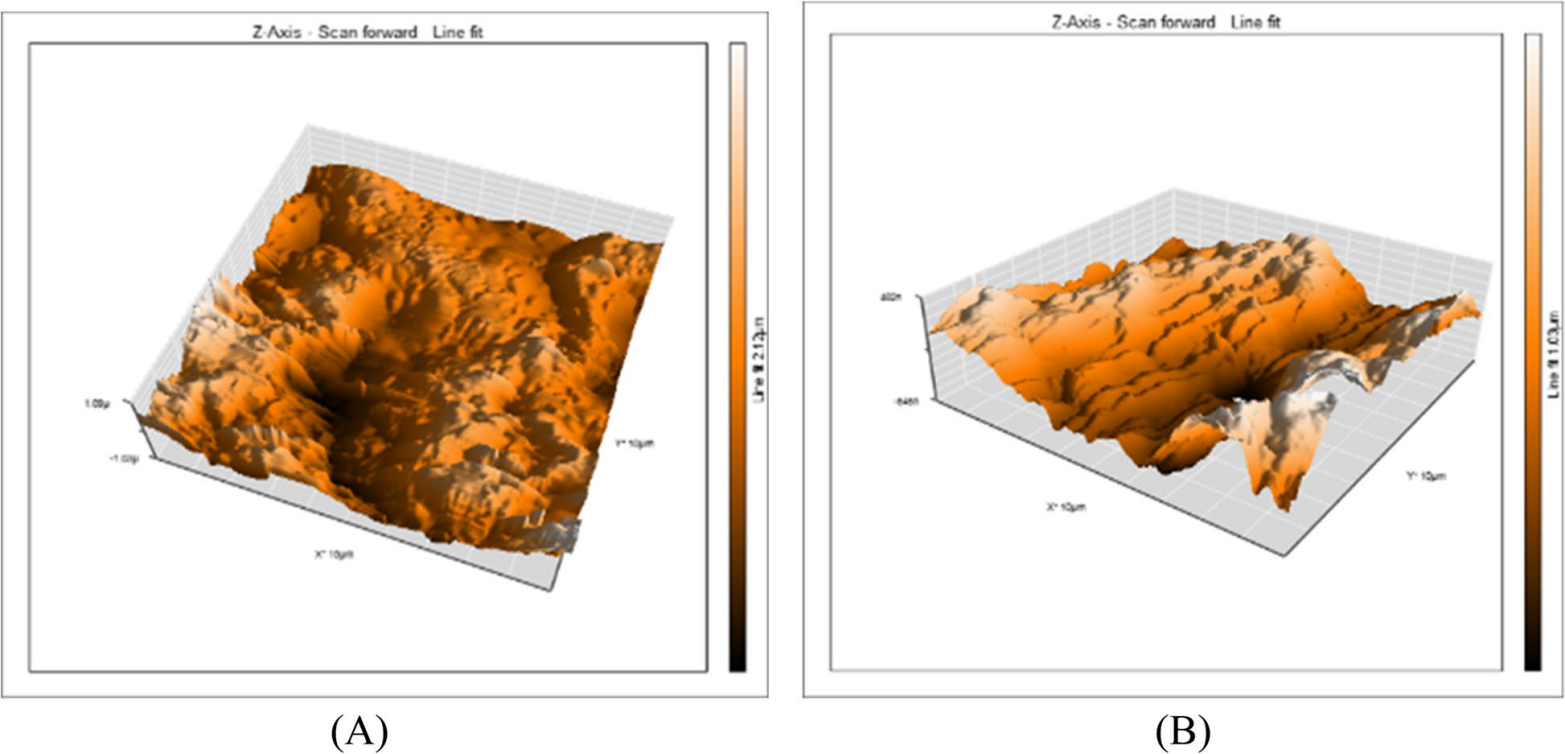
Three-dimension AFM contact mode micrographs of GIC after 24 hours (**A**) and 6 months (**B**) of restoration placement

**Fig. 8:**
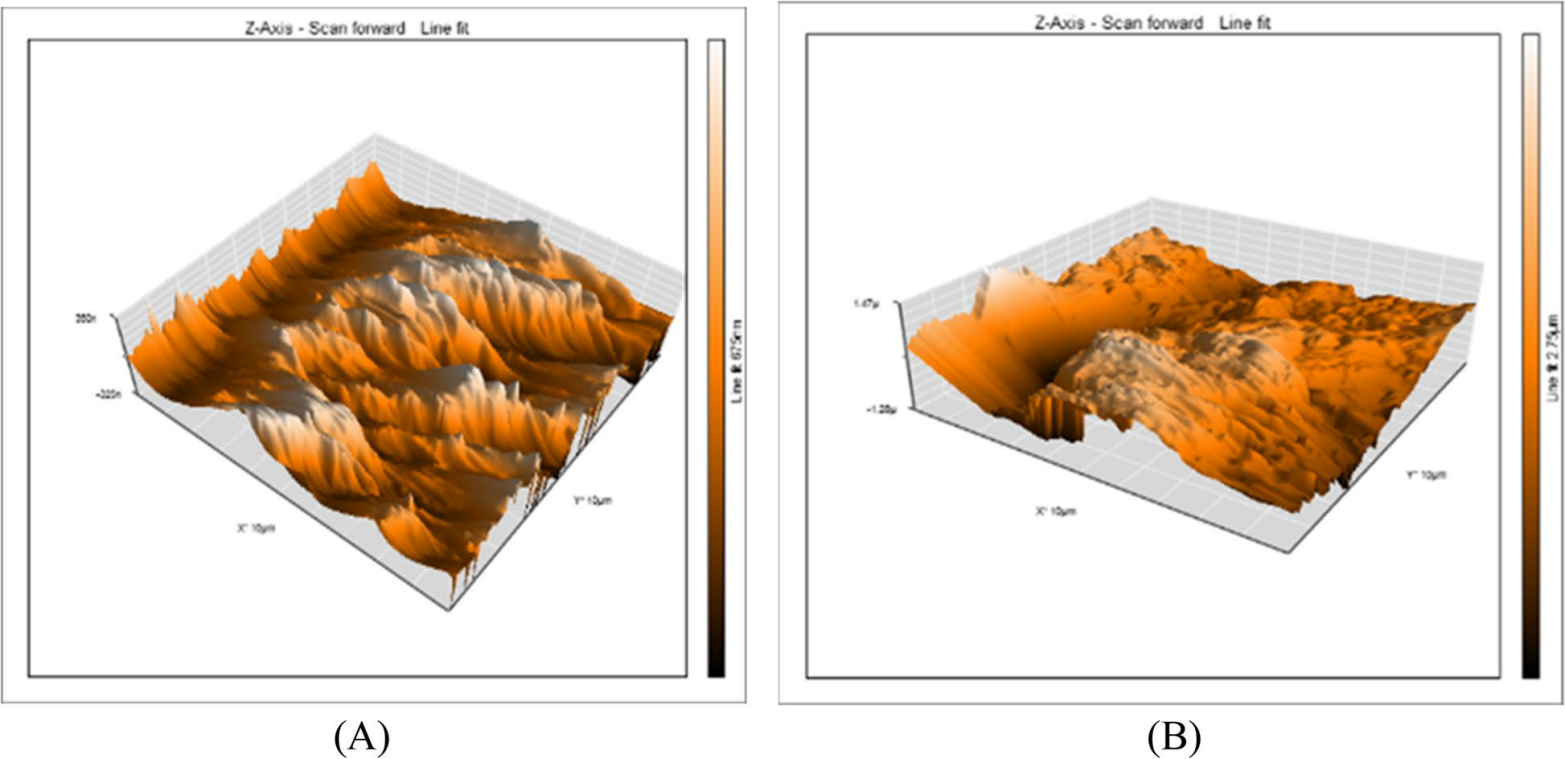
Three-dimension AFM contact mode micrographs of CGIC after 24 hours (**A**) and 6 months (**B**) of restoration placement

**Fig. 9:**
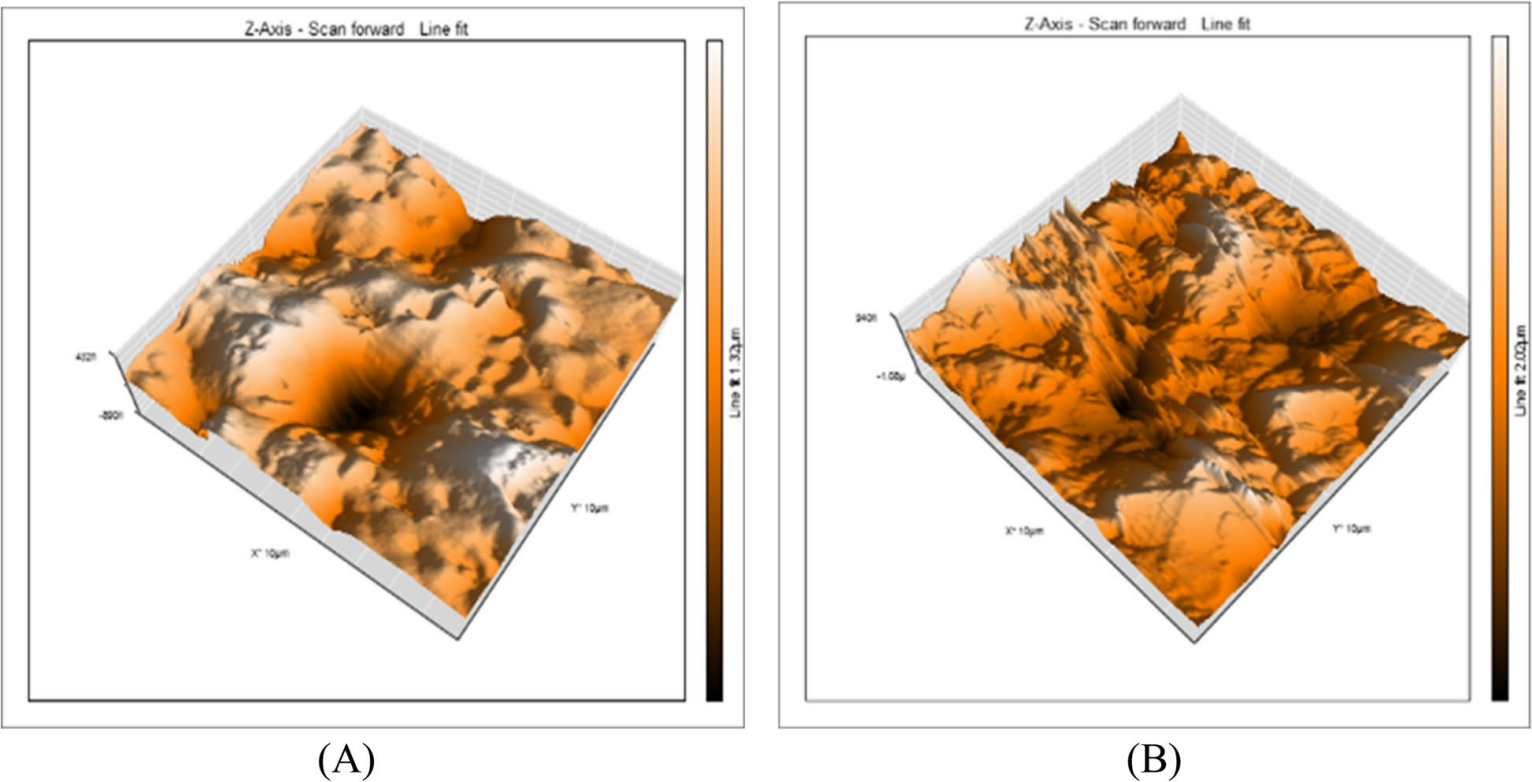
Three-dimension AFM contact mode micrographs of VGIC after 24 hours (**A**) and 6 months (**B**) of restoration placement

A 3D surface analyzer device was utilized to qualitatively evaluate surface roughness in this research. Every specimen is given 3D and histogram images with (Ra) measurements from the 3D surface analyzer system. The histogram’s Y axis shows the total number of times each reading was taken (Mm), and the resulting (Ra) value was calculated as the average of the top five most-read passages. Representative 3D and histogram microscopy for each subgroup of RMGIC, CRMGIC, VRMGIC, GIC, CGIC, VGIC are presented in Figs. 4, 5, 6, 7, 8 and 9 respectively.

Quantitative data were described using mean ± Standard deviation for normally distributed data after testing normality using Shapiro Wilk test. Significance of the obtained results was judged at the (≤ 0.05) level. One Way ANOVA test was used to compare more than 2 independent groups with Post Hoc Tukey test to detect pair-wise comparison. Two Way ANOVA test was used to study the combined effect of 2 independent factors on dependent continuous outcome with estimation of R2. Paired t test was used to assess difference between pre and post treatment values.

### Fluoride specific ion electrode test

Shapiro-Wilk test showed normal distribution pattern of all values (*p* > 0.05). A descriptive statistic was calculated in the form of mean and standard deviation (SD). Then, the significance of difference between groups was tested using Two-way ANOVA to compare between the groups. The Least Significant Difference (LSD) Post-hoc test was used to detect difference between each group, and showed that SDI GIC after 14 days of measurement had the highest mean of fluoride release (36.38 ± 3.16 PPM) and SDI RMGIC after 30 days of measurement had the second highest mean of fluoride release (43.28 ± 1.89 PPM), which were significant. Tukey post-hoc test was used for multiple comparisons, and showed that there was a significant difference between all groups as presented in Table 3.

**Table 3:** Comparison between RMGIC, SDI RMGIC, VRMGIC, GIC, SDI GIC and VGIC groups of fluoride release in different time of storage

Groups *n* = 5	Time / Mean (PPM)							
	24 h.	7 days	14 days	30 days	45 days	60 days	75 days	90 days
RMGIC	39.48 ± 0.13^d^	33.08 ± 0.14^a^	34.88 ± 0.12^b^	44.48 ± 0.13^e^	35.38 ± 0.13^b^	41.48 ± 0.12^e^	38.28 ± 0.13^d^	42.68 ± 0.14^e^
SDI RMGIC	37.88 ± 0.12^c^	35.88 ± 0.13^b^	32.68 ± 0.14^a^	43.28 ± 1.89^e^	32.16 ± 0.16^a^	41.78 ± 0.13^e^	38.08 ± 0.14^d^	43.38 ± 0.12^e^
VRMGIC	38.38 ± 0.14^d^	33.58 ± 0.13^b^	27.88 ± 0.13^a^	35.28 ± 0.14^b^	27.18 ± 0.12^a^	39.38 ± 0.14^d^	37.78 ± 0.12^c^	42.38 ± 0.13^e^
GIC	40.18 ± 0.13^d^	35.48 ± 0.13^b^	32.38 ± 0.14^a^	41.48 ± 0.13^e^	37.68 ± 0.12^c^	41.78 ± 0.13^e^	38.28 ± 0.12^d^	42.48 ± 0.14^e^
SDI GIC	38.28 ± 0.13^d^	34.88 ± 0.12^b^	36.38 ± 3.16^c^	53.68 ± 0.12^f^	57.28 ± 0.13^f^	42.38 ± 0.12^e^	37.68 ± 0.13^c^	43.58 ± 0.12^e^
VGIC	38.38 ± 0.14^d^	32.38 ± 0.13^a^	39.58 ± 0.13^d^	50.88 ± 0.14^f^	39.18 ± 0.12^d^	43.38 ± 0.14^e^	38.98 ± 0.14^d^	43.28 ± 0.13^e^

Groups identified by different superscripts were significantly different at *p* < 0.05

### Micromorphological analysis of the restoration/dentin interface

**Fig. 10:**
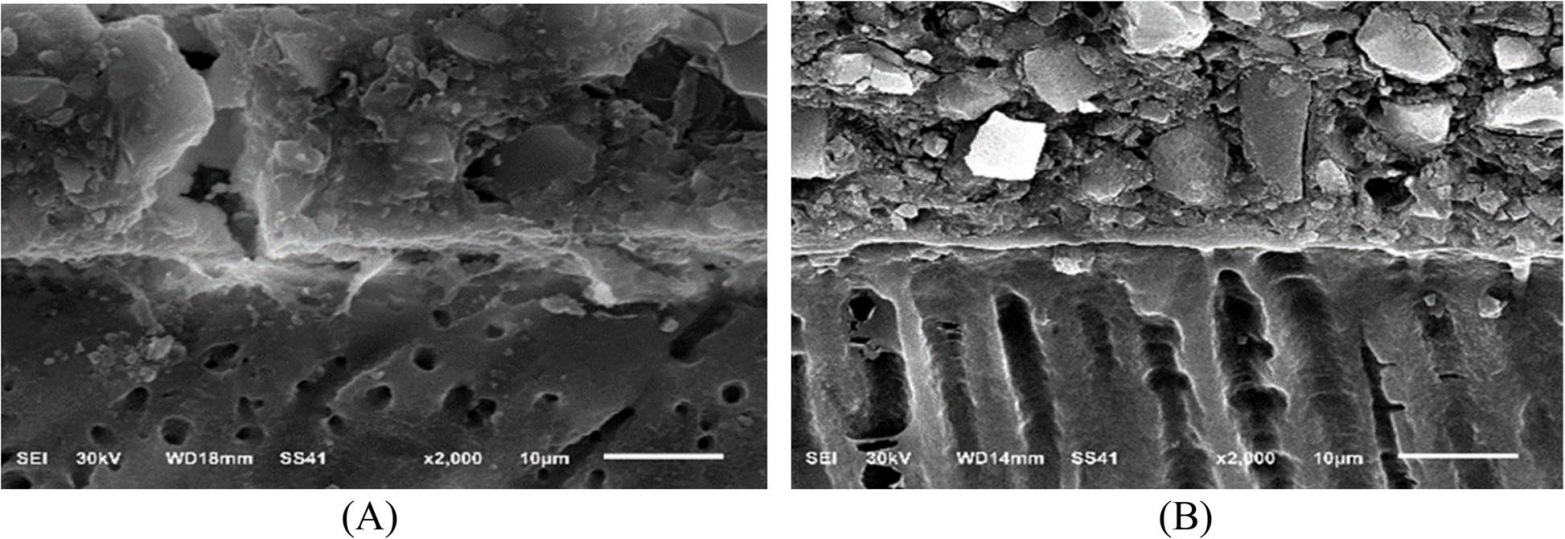
SEM micrographs of the restoration/dentin interface of RMGIC treated with acid/base challenge method at X2000 magnification. **A** after 24 hours of restoration placement. The micrographs are showing acid-base resistant layer (ABR) at the interface between RMGIC and the dentin. with no signs of separation throughout the interface. **B** after 6 months of restoration placement. The micrographs are showing acid-base resistant layer (ABR) at the interface between RMGIC and the dentin. with no signs of separation throughout the interface

**Fig. 11:**
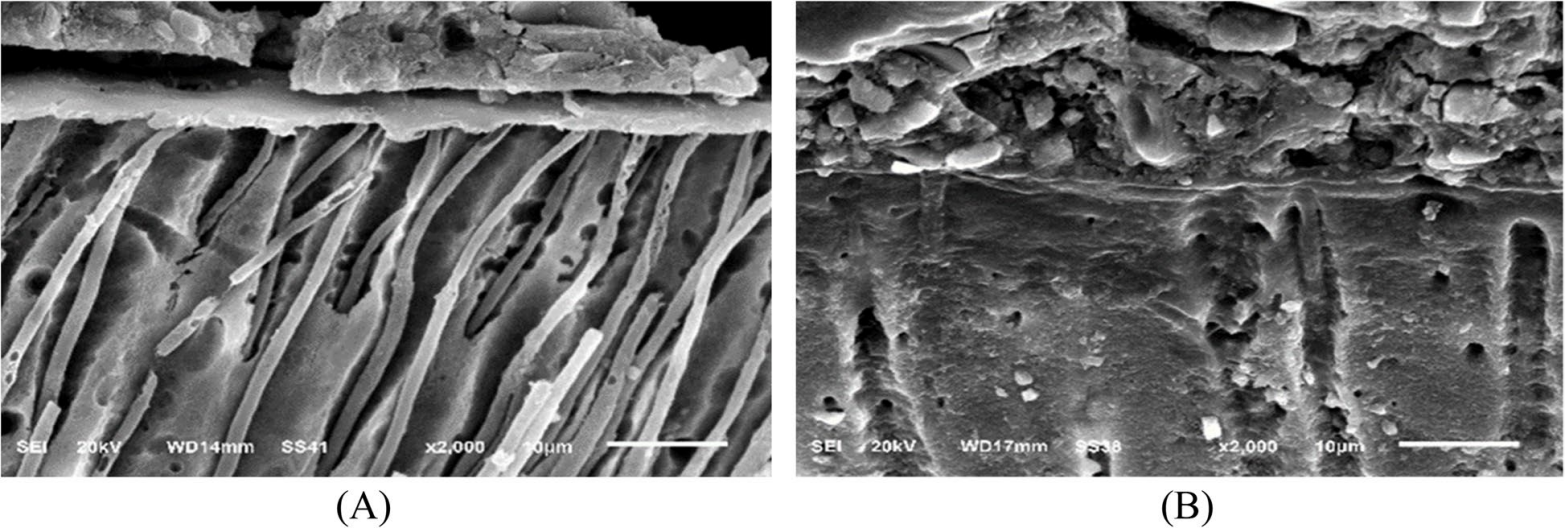
SEM micrographs of the restoration/dentin interface of CRMGIC treated with acid/base challenge method at X2000 magnifications. **A** after 24 hours of restoration placement. The micrographs are showing acid-base resistant layer (ABR) at the interface between CRMGIC and the dentin. numerous thick and long cylindrical-shaped resin tags with signs of separation throughout the interface. **B** after 6 months of restoration placement. The micrographs are showing acid-base resistant layer (ABR) at the interface between CRMGIC and the dentin. with no signs of separation throughout the interface

**Fig. 12:**
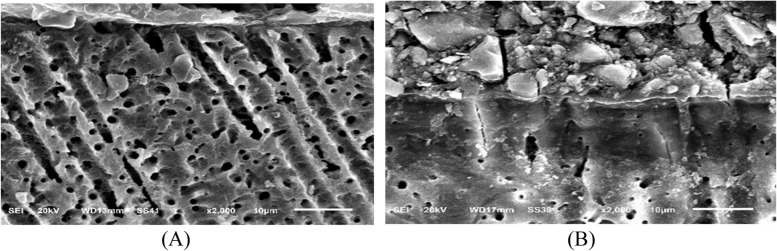
SEM micrographs of the restoration/dentin interface of VRMGIC treated with acid/base challenge method placement at X2000 magnifications. First row: after 24 hours of restoration. The micrographs are showing acid-base resistant layer (ABR) at the interface between RMGIC and the dentin. with signs of separation throughout the interface. Second row: after 6 months of restoration placement. The micrographs are showing acid-base resistant layer (ABR) at the interface between VRMGIC and the dentin. with no signs of separation throughout the interface

**Fig. 13:**
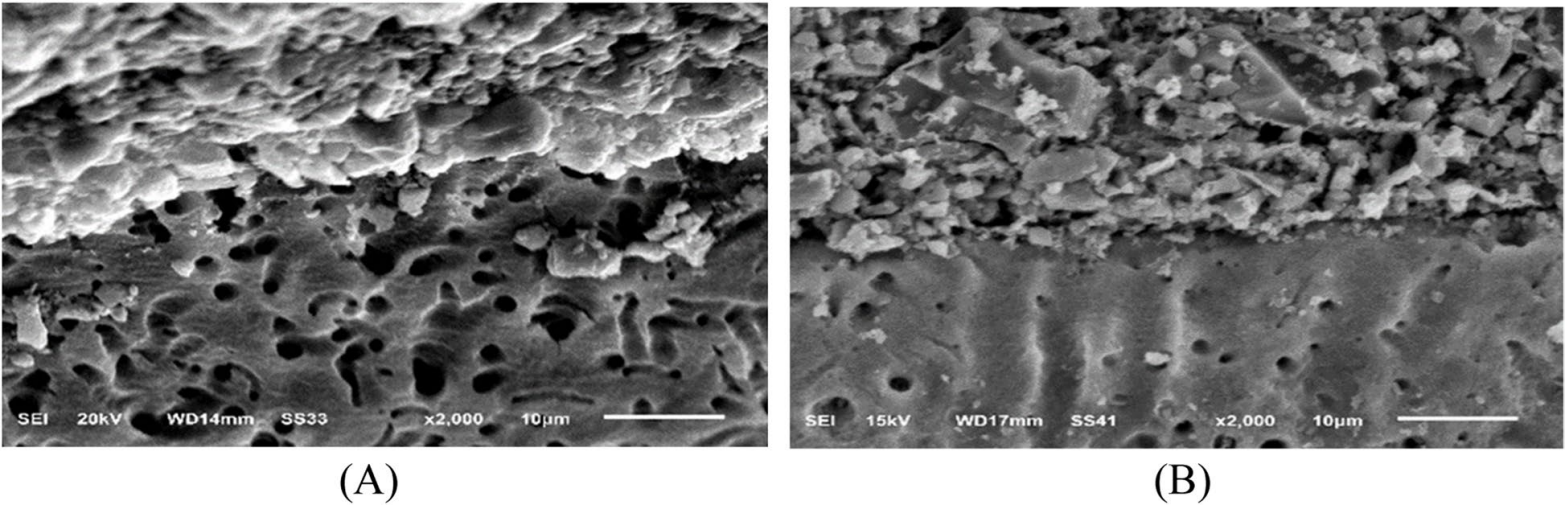
SEM micrographs of the restoration/dentin interface of GIC treated with acid/base challenge method at X2000 magnifications. **A** after 24 hours of restoration placement. The micrographs are showing acid-base resistant layer (ABR) at the interface between GIC and the dentin. with no signs of separation throughout the interface. **B** after 6 months of restoration placement. The micrographs are showing acid-base resistant layer (ABR) at the interface between GIC and the dentin. with no signs of separation throughout the interface

**Fig. 14:**
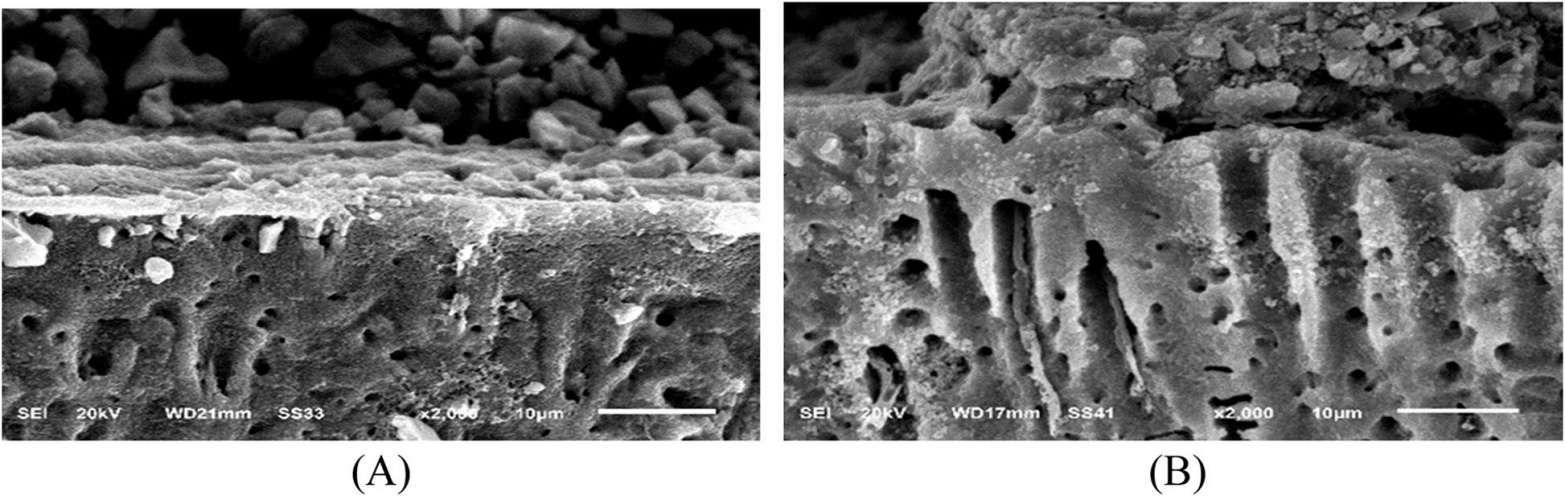
SEM micrographs of the restoration/dentin interface of CGIC treated with acid/base challenge method at X2000 magnifications. **A** after 24 hours of restoration placement. The micrographs are showing acid-base resistant layer (ABR) at the interface between CGIC and the dentin. with signs of separation throughout the interface. **B** after 6 months of restoration placement. The micrographs are showing acid-base resistant layer (ABR) at the interface between CGIC and the dentin. with signs of separation throughout the interface

**Fig. 15:**
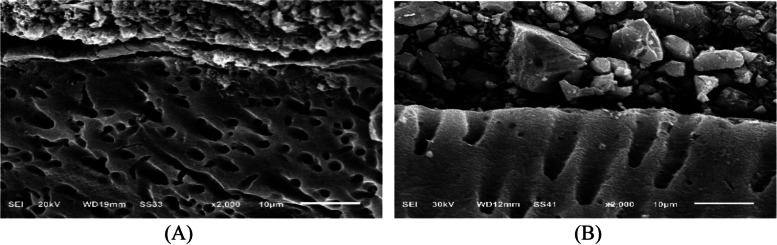
SEM micrographs of the restoration/dentin interface of VGIC treated with acid/base challenge method at X2000 magnifications. **A** after 24 hours of restoration placement. The micrographs are showing acid-base resistant layer (ABR) at the interface between VGIC and the dentin. With signs of separation throughout the interface. **B** after 6 months of restoration placement. The micrographs are showing acid-base resistant layer (ABR) at the interface between VGIC and the dentin. with signs of separation throughout the interface

The SEM micrographs of the restoration/dentin interface of RMGIC, CRMGIC, VRMGIC, GIC, CGIC and VGIC are shown in sequence (Figs. 10, 11, 12, 13, 14 and 15 respectively) at X2000 magnification, respectively. All the tested specimens (acid/base challenge) were examined. RMGIC group showed in acid/base challenge method after 24 h of restoration placement. The micrographs demonstrated an acid-base resistant layer (ABR) at the RMGIC-dentin interface. with no evidence of separation along the interface, after 6 months of restoration placement. The micrographs showed acid-base resistant layer (ABR) at the interface between RMGIC and the dentin. with no signs of separation throughout the interface.

CRMGIC group showed in the acid/base challenge method after 24 h of restoration placement Micrographs demonstrated an acid-base resistant layer (ABR) at the interface between CRMGIC and dentin. Numerous thick and long cylindrical-shaped resin tags with signs of separation throughout the interface, after 6 months of restoration placement. The micrographs showed acid-base resistant layer (ABR) at the interface between CRMGIC and the dentin. with no signs of separation throughout the interface.

VRMGIC group showed in the acid/base challenge method after 24 h of restoration. Micrographs demonstrate an acid-base resistant layer (ABR) at the interface between RMGIC and dentin. with separation signs throughout the interface, after 6 months of restoration placement. The micrographs showed acid-base resistant layer (ABR) at the interface between VRMGIC and the dentin. with no signs of separation throughout the interface.

GIC group showed in the with acid/base challenge method after 24 h of restoration placement. The micrographs depict an acid-base resistant layer (ABR) at the interface of GIC and dentin with no signs of separation throughout the interface, after 6 months of restoration placement. The micrographs showed acid-base resistant layer (ABR) at the interface between GIC and the dentin. with no signs of separation throughout the interface.

CGIC group showed in acid/base challenge method after 24 h of restoration placement The micrographs demonstrated an acid-base resistant layer (ABR) at the CGIC-dentin interface with signs of separation throughout the interface, after 6 months of restoration placement. The micrographs showed acid-base resistant layer (ABR) at the interface between CGIC and the dentin. with signs of separation throughout the interface.

VGIC group showed in the acid/base challenge method after 24 h of restoration placement The micrographs demonstrated an acid-base resistant layer (ABR) at the VGIC-dentine interface. Throughout the interface, there are indicators of separation, after 6 months of restoration placement. The micrographs showed acid-base resistant layer (ABR) at the interface between VGIC and the dentin. with signs of separation throughout the interface.

## Discussion

The introduction of glass ionomer cements serves the need for a substance that adheres to the tooth’s substructure without causing significant damage to the pulp. Hydroxapatite is composed of calcium ions, and GIC can form chemical bonds with these ions. This material can be used to bond healthy or caries-damaged dentin, despite its inferior cohesive strength when compared to resin-based composite [1] To resolve the limitations of conventional glass ionomer cements (GICs), resin-modified glass ionomer cements (RMGIC) were created as an alternative. RMGICs are typically composed of 20% light-cured methacrylates and 80% GIC components (fluoroaluminosilicate glass and polyacrylic acid). Within twenty-four hours, RMGICs will be entirely cured without needing to be exposed to a curing light. This distinguishes RMGIC from other composite materials based on polyacid-modified polymers (such as compomer and giomer) [10]. 

The primary components of GICs are a powdered form of fluoro-aluminosilicate glass and an aqueous solution of polyalkenoic acids, which are carboxylic acids. The majority of the aqueous fraction is polyacrylic acid [6]. GICs are mainstream restorative materials that are bioactive and have a wide range of uses such as lining, bonding, sealing, luting or restoring a tooth. GICs are useful for reasons of adhesion to tooth structure, fluoride leaching and being tooth-colored, although their sensitivity to moisture, inherent opacity, long-term wear and strength are not as adequate as desired [11]. Within twenty-four hours of mixing, GIC undergoes a setting reaction that renders it susceptible to moisture and temperature fluctuations. If exposed to moisture, premature GIC may experience component loss, surface wear, and diminished translucency. However, when the reaction occurs in dry conditions, the GIC is more likely to lose water, which compromises adhesion, alters the material’s dimensions, and causes internal cracks, thereby decreasing its strength. To combat this early susceptibility to moisture, the surface of GIC is coated with protective materials such as varnishes, adhesive systems, petroleum jelly, and nanofilled self-adhesive light-cured coating [12]. So, despite its limitations, this study attempted to explain the influence of different types of coats on surface roughness, fluoride release, and interference between restoration and tooth from modern resin-modified and standard glass ionomer restorations.

Surface roughness measured by using atomic force microscopy (AFM). The AFM works by having a cantilever tip physically interact with molecules on the cell surface. Cantilever deflections are used to measure adhesion forces exerted by molecules on a cell’s surface [13]. Depending on the variation in micro-surface height, the gravitational or repulsive force between the tip and the sample surface will change during the scanning process. Tracing the up-and-down motion of the tip will provide information about the sample’s surface topography [14] Scanning electron microscope used to obtain and quantitative data of tooth structure, which explaining the complex between restoration-tooth interface [15]. Fluoride ions specific test has many advantages include simplicity, accuracy, speed, and elimination of ashing, distillation, or diffusion steps which previously were needed to separate fluoride from interfering ions [16]. 

The result of AFM in this study showed that there is significant difference in surface roughness between tested groups, in all coated groups surface roughness decreased during the first 24 h, while uncoated groups appeared increase in surface roughness within first 24 h, this show the ability of different type of coats to protect the GIC and RMGIC restorations from been contaminated with moisture during the first 24 h.so the result agreed with Iman et al. [17] study which concluded that uncoated, brushed specimens submerged in ethanol had the greatest mean surface roughness value. Specimens coated in unbrushed copal varnish and submerged in ethanol had the smoothest average surface. Regarding the fluoride release test, all groups demonstrate constant fluoride leakage during 90 days of storage, despite the fact that measurements were made at different times during storage. This study show there is no effect of coats on fluoride release which is disagree with Kelic et al. [18] and Valentina et al. [19] whom highlighted that different coats had different effects on fluoride release from the studied restorative materials, and when compared to varnish-coated and uncoated samples the amount of released fluoride was drastically lower in those covered with a nano filled surface coating agent, respectively .

Regarding the micromorphological analysis, all tested groups showed relatively similar results, acid-base resistant layer was observed in all groups which is indication of the ability to resist demineralization. hybrid layer was identified between the dentin and restoration. CRMGIC group which showed numerous thick and long cylindrical-shaped resin tags in the acid-base challenge method after 24 h of restoration placement, while after 6 months resin tags were absence. The presence or absence of resin tags in the specimens tested suggests that resin tags are not responsible for the interference. Therefore, there is no relation between the absence or presence of resin tags and the interference between tooth and restoration. The result of this study show there is no effect of coating on tooth-restoration interference. The findings of the micromorphological analysis in this study were in agreement with Sobh et al. [5] all tested materials showed some ability to resist demineralization at the restoration margins. In light of the results of this study, the first null hypothesis, which states that there is no significant effect of different surface coating methods on surface roughness was rejected, Also, the second null hypothesis which stated that there is no significant effect of different surface coating methods on fluoride release was rejected.

### Limitations of study

Considering this is an in vitro study, it is limited by laboratory conditions. Lack of masticatory loading, dentin fluid, pulpal pressure, pH change, and saliva buffering are some of the limiting factors. Furthermore, due to a lack of financial means to fund the research, the study had a small sample size. As well as the relatively short storage period may have prevented a comprehensive evaluation of the coat’s performance over time.

## Conclusions

Based on the results of this study, various coatings enhance surface roughness in the initial 24 h of restoration installation by reducing or preventing moisture-contamination. Different coat types seem that have no influence on fluoride release and the micromorphological features of the restoration/dentin interface exhibited approximately similar results in the coat and uncoated groups.

## Data Availability

All data generated and analyzed in this review are included within the article.
